# Stimulation of Dopamine D4 Receptors in the Nucleus Accumbens Shell Increases Palatable Food Intake in Satiated Male Rats: Modulation by NMDA and AMPA Receptors

**DOI:** 10.3390/brainsci14111103

**Published:** 2024-10-30

**Authors:** Refugio Cruz-Trujillo, Daniel Díaz-Urbina, José Alfredo Díaz-Gandarilla, Dolores Guadalupe Vidal-López, Rodrigo Erick Escartín-Pérez, Juan Manuel Mancilla-Diaz, Benjamín Florán, Juan Gabriel Tejas-Juárez

**Affiliations:** 1 Escuela de Ciencias Químicas, Universidad Autónoma de Chiapas (UNACH), Carretera Panamericana Ocozocoautla-Cintalapa Km. 2.5, Ocozocoautla de Espinosa 29140, Mexico; refugio.cruz@unach.mx; 2 Departamento de Químicos Farmacobiólogos, Universidad Pablo Guardado Chávez (UPGCH), Libramiento Norte Oriente No. 3450, Tuxtla Gutiérrez 29040, Mexico; 3 Laboratory on Neurobiology of Compulsive Behaviors, NIMH, National Institutes of Health, Bethesda, MD 20892, USA; daniel.diaz-urbina@nih.gov; 4 Laboratorio de Neurobiología de la Alimentación, Grupo de Investigación en Nutrición, División de Investigación y Posgrado de la FES Iztacala, UNAM, Ciudad de México 04510, Mexico; escartin@unam.mx (R.E.E.-P.); jmmd@unam.mx (J.M.M.-D.); 5 División Académica Multidisciplinaria de Comalcalco, Universidad Juárez Autónoma de Tabasco (UJAT), Comalcalco 86658, Mexico; alfredo.diaz@ujat.mx; 6 Universidad de Ciencias y Artes de Chiapas (UNICACH), Tuxtla Gutiérrez 29014, Mexico; lolita.vidal@unicach.mx; 7 Centro de Investigación y de Estudios Avanzados del IPN, Departamento de Fisiología, Biofísica y Neurociencias, Ciudad de México 07360, Mexico; bfloran@fisio.cinvestav.mx

**Keywords:** dopamine D4 receptor, NMDA receptor, AMPA receptor, nucleus accumbens shell, food intake, palatability, satiated rats

## Abstract

Background/Objectives: Palatability significantly influences food consumption, often leading to overeating and obesity by activating the brain’s reward systems. The nucleus accumbens (NAc) plays a central role in this process, modulating reward mechanisms primarily via dopamine through D2-like receptors (D2R, D3R, D4R). While the involvement of D2 receptors in feeding is well-documented, the role of D4 receptors (D4Rs) is less clear. Methods: Male Wistar rats received intra-NAc shell microinjections of the D4R agonist PD-168077 and the antagonist L-745870. This study also examined the modulation between D4R and glutamatergic transmission by administration of NMDA, NMDA receptor antagonist AP-5, AMPA, and AMPA receptor antagonist CNQX. Results: PD-168077 increased sweet solution intake by 46%, an effect that was reversed by L-745870. Pre-treatment with NMDA prevented the stimulatory effect of PD-168077, whereas the NMDA receptor antagonist AP-5 had no such effect. Additionally, AMPA administration reduced sweet solution intake by 63%, counteracting the effect of PD-168077, while the AMPA receptor antagonist CNQX, on its own, increased intake by 40%. Conclusions: These findings suggest that D4Rs promote hedonic feeding by modulating glutamatergic transmission in the NAc shell, highlighting the complexity of D4R involvement in food intake regulation. This study underscores the potential of targeting D4Rs for therapeutic interventions in eating disorders and obesity, though further research is essential to clarify the precise mechanisms through which D4R modulates AMPA and NMDA receptor activity in feeding behavior.

## 1. Introduction

Palatability is a crucial factor influencing food intake, often determining meal size and enhancing food reward. Both experimental and clinical evidence have shown that highly palatable foods and sugar solutions can drive overconsumption, even in the absence of hunger, eventually contributing to obesity and eating disorders such as binge eating.

The brain’s reward systems mediate motivation by integrating sensory cues, including sight, smell, and taste [1]. Key brain regions involved in this process include the ventral tegmental area (VTA), lateral hypothalamus, orbitofrontal cortex, central amygdala, hippocampus, insula, and, notably, the nucleus accumbens (NAc) [2]. Among these areas, the NAc plays a pivotal role in modulating reward-driven behaviors. Experimental studies have demonstrated that electrical stimulation of the NAc inhibits sucrose licking in rodents [3]. In humans, obesogenic diets have been shown to impair the functioning of brain reward centers, including the NAc [4].

The NAc plays a pivotal role in modulating hedonic food intake and motivation. It receives mesolimbic dopaminergic inputs from the ventral tegmental area (VTA) [5,6,7]. Experimental evidence shows that the consumption of palatable foods increases dopamine release in the NAc, even in satiated rats [8]. Additionally, clinical studies have demonstrated that eating palatable foods elevates dopamine levels in the dorsal striatum, which correlates with self-reported feelings of pleasure [2]. Thus, dopaminergic transmission in the NAc is crucial for promoting brain reward, hedonic eating, and even drug addiction [8,9].

Dopamine exerts its effects through five receptor subtypes (D1R–D5R), all part of the G-protein-coupled receptor superfamily. Three of these—D2R, D3R, and D4R—belong to the D2-like receptor family, which inhibits adenylate cyclase activity [10]. While the role of D2R in regulating eating behavior and energy homeostasis has been extensively studied [11,12,13], the specific function of D4 receptors (D4R) in controlling food intake remains underexplored. Some studies suggest that blocking D4Rs increases food intake and meal size, impairing the satiety response, which may contribute to obesity risk [14,15,16]. Conversely, more recent evidence indicates that administering the selective D4R antagonist L-745,870 centrally or directly into the NAc reduces sucrose consumption and lever pressing for sucrose in rats [17,18]. These conflicting findings underscore the complexity of D4R’s role in regulating food intake and motivation, highlighting the need for further research to clarify the underlying mechanisms.

D4 receptors (D4R) are expressed in several brain areas, including glutamatergic pyramidal neurons and GABAergic interneurons in the prefrontal cortex, the paraventricular nucleus of the hypothalamus (PVN), the reticular substantia nigra, and NAc, among others [19,20,21]. The medium spiny neurons (MSNs) of the NAc receive dense glutamatergic inputs from various regions such as the medial prefrontal cortex, ventral subiculum, PVN, and hippocampus. These MSNs co-express glutamatergic AMPA receptors (AMPAR) alongside dopaminergic receptors [22,23,24]. Similar to dopamine, glutamate modulates MSN activity, influencing reward-oriented behavior [25]. Notably, enhanced calcium-AMPAR signaling has been observed in diet-induced obesity (DIO) rats [4].

Research indicates that intra-NAc injections of AMPA/kainate agonists increase food intake, while blocking N-methyl-D-aspartate receptors (NMDAR) does not affect feeding behavior [26]. Additionally, antagonizing AMPAR in the NAc shell has been shown to increase food intake in ad libitum-fed rats, whereas activating these receptors reduces the sucrose-evoked breakpoint and lever pressing in rats subjected to short-term food deprivation (2 h) [27]. Furthermore, direct stimulation of AMPAR within the NAc shell suppresses both deprivation-induced feeding and the intake of a palatable 5% sucrose solution [28].

Given that D4 receptors (D4R) are expressed in multiple brain areas involved in food motivation, including the NAc shell, and may influence presynaptic glutamate release [20,29,30], it is postulated that D4R activation could reduce AMPAR and NMDAR activity, thereby promoting hedonic feeding, similar to the effects observed with AMPAR and NMDAR blockade [27,28]. To test this hypothesis, we aimed to investigate the effect of a D4R agonist in the NAc shell on palatable food intake in pre-satiated rats, with a specific focus on how D4R modulation impacts AMPAR and NMDAR activity.

## 2. Materials and Methods

### 2.1. Animals

Male Wistar rats, weighing 220–240 g (8 weeks old), were provided by the FES Iztacala vivarium at the Universidad Nacional Autónoma de México (UNAM). The animals were individually housed in acrylic home cages (27 × 37 × 15 cm) under a 12 h light/dark cycle (lights on at 08:00 a.m.) and maintained at a controlled room temperature of 22 ± 2 °C. During the 1-week acclimation period, the rats had unrestricted access to water and standard chow pellets.

All experiments complied with the ethical guidelines in the Technical Specifications for the Production, Care, and Use of Laboratory Animals as established in the Official Mexican Norm for Animal Care (NOM-062-ZOO-1999) and were approved by the Ethical Committee of FES Iztacala UNAM (CE/FESI/112017/1221). These guidelines were strictly followed to ensure the ethical treatment and welfare of the laboratory animals throughout the study.

### 2.2. Diet

The animals were fed standard laboratory chow (26.6% protein, 16.5% fat, and 56.8% carbohydrates, LabDiet^®^ 5008, Lab suply, Richmond, IN, USA), tap water, and a sweetened mixture of evaporated milk (Carnation^®^, 6% total fat, 3.75% saturated fat, 9.5% carbohydrates, 6% protein) blended with 10% sucrose.

### 2.3. Drugs

All drugs were prepared in sterile 0.9% saline to achieve the desired concentrations. Fresh solutions were made before each injection to ensure precise and consistent dosing: the appropriate quantity of each drug was measured using an analytical balance and transferred to a 1.5 mL Eppendorf Tube^®^. The drug was then dissolved in sterile 0.9% saline solution and mixed with a vortex mixer until the desired concentration was reached. The following drugs were obtained from Tocris Bioscience (Atlantic Road, Bristol, UK): PD-168077 (Code: 1065; N-[[4-(2-cyanophenyl)-1-piperazinyl]methyl]-3-methylbenzamide maleate salt), L-745870 (Code: 1002; 3-(4-[4-chlorophenyl]piperazin-1-yl)-methyl-1H-pyrrolo [2,3-b]pyridine trihydrochloride), D-AP-5 (Code: 0106; D-(-)-2-amino-5-phosphonopentanoic acid), and AMPA (Code: 1074; (RS)-α-amino-3-hydroxy-5-methyl-4-isoxazolepropionic acid hydrobromide). The following drugs were sourced from Sigma-Aldrich Química (Parque Industrial Toluca 2000, Toluca, México): NMDA (Code: M3262; (R)-2-(methylamino)succinic acid) and CNQX (Code: C239; 6-cyano-7-nitroquinoxaline-2,3-dione disodium salt hydrate).

### 2.4. Stereotaxic Surgery

Male Wistar rats were deeply anesthetized using a ketamine/xylazine combination (Laboratorios Aranda/PiSA Agropecuaria, Mexico; 112.5/22.5 mg/kg, intraperitoneally). The rats were then securely positioned in a stereotaxic apparatus to facilitate precise and reproducible surgical procedures. A stainless-steel guide cannula (23 G, 15 mm long, BD Precision Glide, Becton Dickinson and Co., Franklin Lakes, NJ, USA) was unilaterally implanted into the overlying region of the NAc shell, with stereotaxic coordinates of anteroposterior (AP) +1.52 mm, medial–lateral (ML) ± mm from bregma, and dorsal–ventral (DV) −6.0 mm relative to the dura mater. The cannula was secured to the skull using a stainless-steel screw and dental acrylic cement. To prevent bacterial infections, rats received a subcutaneous injection of enrofloxacin^®^ (2.5 mg/kg) immediately after surgery and again 72 h post-surgery. After a recovery period of 7 days with ad libitum access to chow and water, the experimental phase commenced [31].

### 2.5. Feeding Paradigm

After the recovery period, rats were subjected to a 22 h period of food deprivation and then trained on a food-restricted feeding program. Over the course of two weeks, the rats were acclimatized to a restricted feeding schedule consisting of 1 h of access to a standard diet (with water) followed by 1 h of access to a palatable solution (without water) during the light phase of the light/dark cycle. This feeding paradigm ensured that the animals were pre-satiated before consuming the palatable food, allowing them to ingest the palatable solution based on its appealing properties [32].

### 2.6. Experimental Procedure

Following the training sessions, rats were randomly assigned to independent groups, and injections of different treatments were administered into the intra-NAc shell after 1 h of access to standard food. Each treatment was infused in a volume of 0.5 µL at a rate of 0.25 µL per minute using a 30 G injector that extended 0.3 mm beyond the guide cannula. The injector remained in place for an additional minute after the injection to ensure adequate diffusion of the drugs.

**Figure 1:**
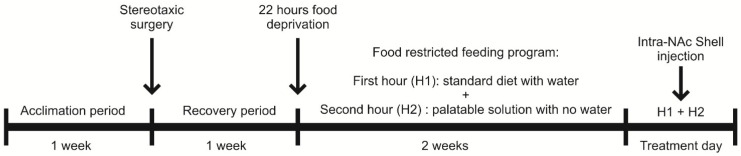
Study schematic overview: Rats were acclimated in individual cages for one week with free access to food and water. After acclimation, stereotaxic surgery was performed. Following a one-week recovery period with ad libitum food and water, a 22 h food restriction protocol was initiated. Rats were then given 1 h of access to standard food followed by 1 h access to palatable food. Drug administration was conducted before the palatable food access period.

All animals received two intra-NAc shell injections: either vehicle/vehicle, vehicle/drug, or drug-1/drug-2. The injections were performed as described, with a 10 min interval between the two administrations. Behavioral tests commenced 10 min after the second injection, with animals placed back in their home cages, where they had access to pre-weighed palatable food without water. At the end of the observation period, the palatable food was removed, and consumption was measured in grams. The meticulous preparation and administration of the drugs were conducted to ensure the accuracy and reproducibility of the experimental procedures while minimizing potential confounding factors in the study (Figure 1). Immediately after the 60 min access to the sweet solution, rats were sacrificed and brains were extracted to verify the correct position of the cannula.

### 2.7. Experimental Design to Evaluate the Modulation of Palatable Solution Consumption by NAc Shell D4R Activation and the Potential Contribution of NMDARs and AMPARs

In the first experiment, we investigated the effect of D4R activation on palatable food intake. Independent groups were randomly assigned to different pharmacological conditions: a control group receiving 0.9% saline, a group receiving D4R activation with PD-168077, a group receiving D4R blockade with L-745870, and a group receiving L-745870 followed by PD-168077.

In experiments 2 and 3, we assessed whether the effects of D4R activation on palatable food intake could be reversed by an NMDAR agonist (NMDA) or antagonist (AP5), respectively, while also evaluating the independent effects of these treatments on palatable food consumption.

Finally, in experiments 4 and 5, we explored the impact of an AMPAR agonist (AMPA) and antagonist (CNQX) on palatable food intake, both in combination with the D4R agonist and when administered independently (see Table 1).

### 2.8. Histology

**Figure 2:**
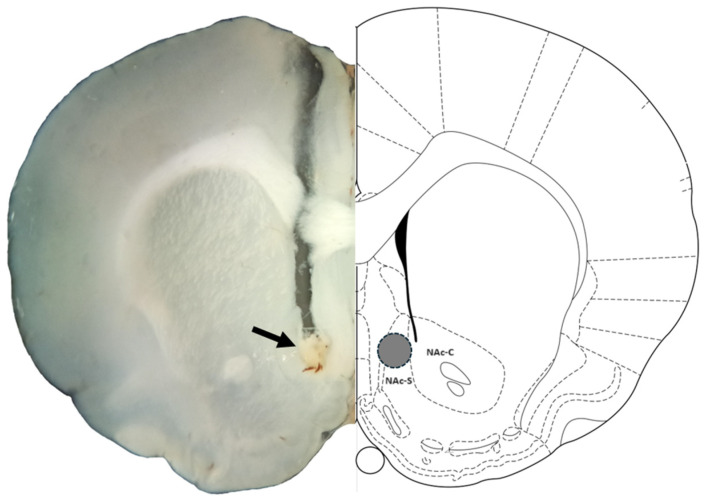
Representative photomicrograph of correct cannula placement in the NAc shell: The left panel displays a coronal brain section of a rat, highlighting the injection site in the nucleus accumbens shell (NAc-S) with an arrowhead marking the representative injection position. The right panel shows a corresponding diagram adapted from the Paxinos and Watson rat brain atlas, where the injection site is indicated by a gray circle within the NAc-S. Data from animals with injections outside the NAc-S, such as those placed in the nucleus accumbens core (NAc-C) or ventral/dorsal to the NAc-S, were excluded from analysis.

After completing the experimental sessions, rats were euthanized with an overdose of sodium pentobarbital and subsequently decapitated. The brains were carefully extracted and placed in a 4% paraformaldehyde solution for 24 h. Coronal brain slices, 200 µm thick, were then prepared using a vibroslicer (Camden Instruments^®^, Leicestershire, UK) and examined under a stereoscope (Figure 2). Data from animals with incorrect cannula placements within the NAc shell were excluded from the statistical analysis.

### 2.9. Data Analysis

Data in this study are presented as mean ± the standard error of the mean (S.E.M.) Following a Kolmogorov−Smirnov normality test, the effects of the drugs on palatable diet consumption (g) were analyzed using one-way analysis of variance (ANOVA). When the ANOVA indicated significance, Dunnett’s post hoc test was applied. A *p*-value of <0.05 was considered statistically significant. Data analysis and visualization were conducted using GraphPad Prism software, version 8.0 (San Diego, CA, USA).

## 3. Results

### 3.1. NAc Shell D4R Modulates Palatable Solution Consumption

**Figure 3:**
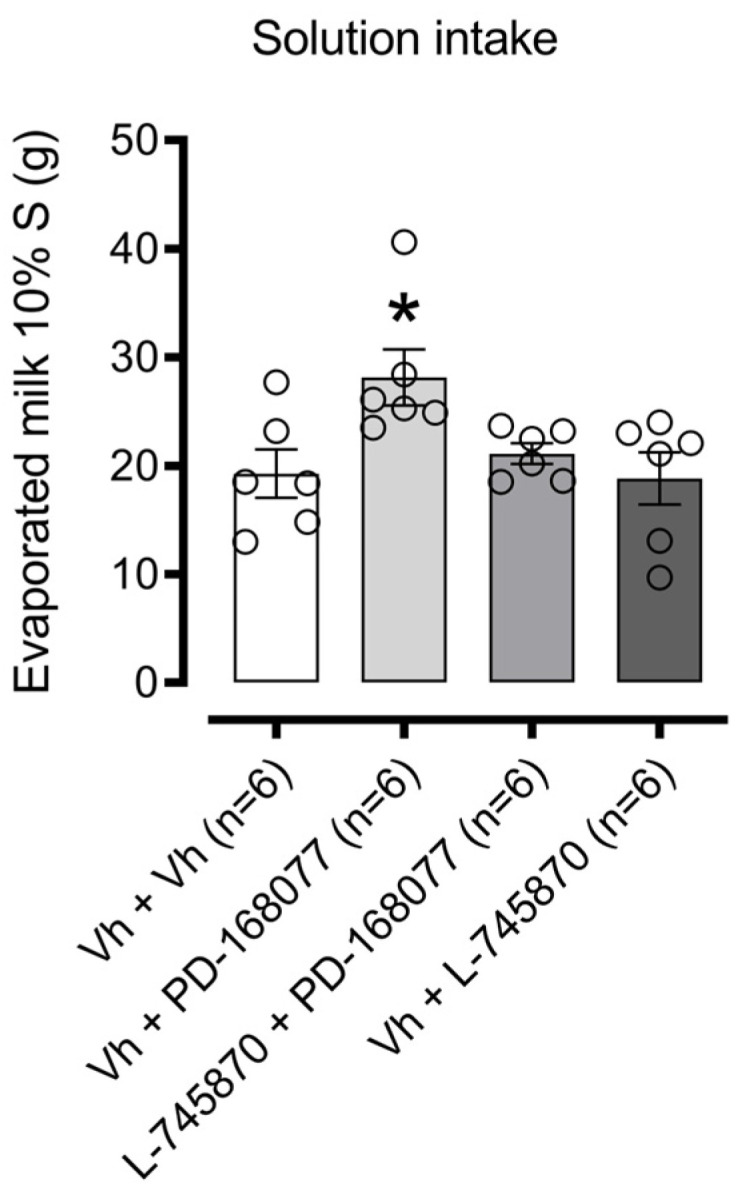
Modulation of palatable solution intake by D4R activation in the NAc shell: Bars represent the mean (±S.E.M.) consumption in grams (g) of a sweet solution prepared with evaporated milk blended with 10% sucrose (S) among groups receiving intra-NAc shell injections of either vehicle (0.9% saline), PD-168077 (0.1 µg), L-745870 (0.1 µg), or a combination pretreatment of L-745870 + PD-168077 in satiated rats. Following a significant one-way ANOVA, Dunnett’s post hoc test was applied, with * *p* < 0.05 indicating significance compared to the vehicle-treated group.

Figure 3 illustrates the effects of intra-NAc shell D4R activation on sweet solution consumption. One-way ANOVA revealed a significant difference in consumption among treatment groups (F(3, 20) = 4.059, *p* = 0.0210). Dunnett’s post hoc analysis showed that the D4R agonist PD-168077 significantly increased palatable solution intake (*p* = 0.0220), an effect reversed by pretreatment with the D4R antagonist L-745870. The antagonist alone, however, had no effect on consumption.

#### 3.1.1. NAc Shell NMDAR Receptor Activation Prevents D4R Effects on Palatable Solution Consumption. The Blockade Did Not Modify Consumption

**Figure 4:**
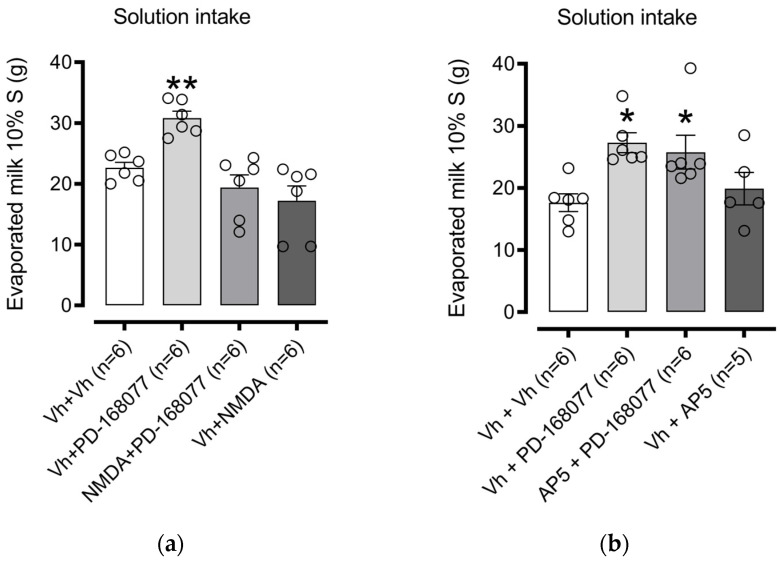
NMDAR activation in the NAc shell blocks D4R-induced increase in palatable solution intake: Bars represent the mean (±S.E.M.) consumption in grams (g) of a sweet solution prepared with evaporated milk blended with 10% sucrose (S) in groups injected in the intra-NAc shell with (**a**) vehicle (0.9% saline), PD-168077 (0.1 µg), NMDA (0.1 µg), and a combination of NMDA + PD-168077 in sated rats; and (**b**) vehicle (0.9% saline), PD-168077, AP-5 (2 µg), and AP-5 + PD-168077. Following a significant one-way ANOVA, Dunnett’s post hoc test was applied, with * *p* < 0.05, ** *p* < 0.01 indicating significance compared to the vehicle-treated group.

We investigated whether the PD-168077-induced increase in consumption depends on glutamatergic signaling in the NAc shell. First, we examined the role of NMDARs. One-way ANOVA revealed significant differences in consumption among treatment groups (F(3, 20) = 11.54, *p* = 0.0001). Dunnett’s post hoc test confirmed that the D4R agonist PD-168077 significantly increased palatable solution intake (*p* = 0.0098), an effect reversed by pretreatment with NMDA (an NMDAR agonist). NMDA alone, however, had no significant effect on consumption (Figure 4a).

Next, we tested the impact of AP-5, an NMDAR antagonist, on solution consumption. One-way ANOVA indicated a significant difference (F(3, 19) = 4.758, *p* = 0.0122). Dunnett’s multiple comparisons showed that PD-168077 increased intake (*p* = 0.0112), and pretreatment with AP-5 did not block the effect of PD-168077 (*p* = 0.0340). AP-5 alone also had no impact on consumption (Figure 4b).

#### 3.1.2. NAc Shell AMPAR Receptor Activation Prevents D4R Effects on Palatable Solution Consumption. The Blockade Modify Consumption

**Figure 5:**
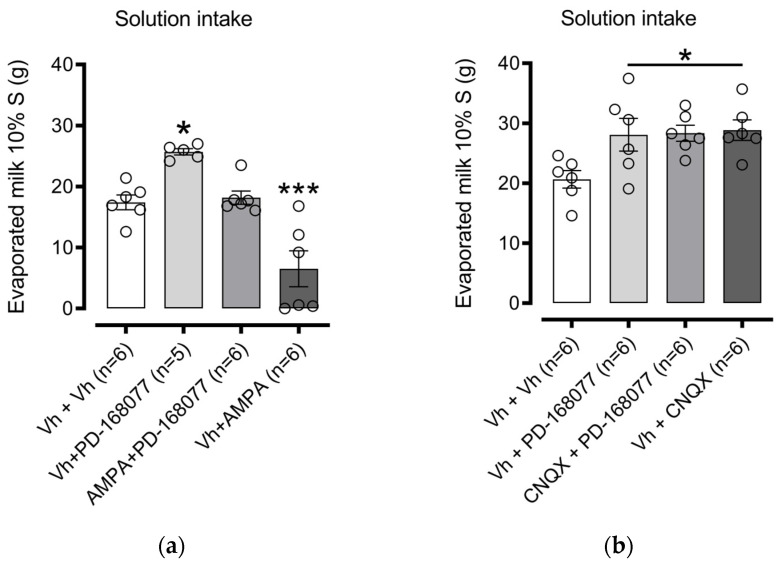
AMPAR Activation in the NAc Shell Reverses D4R-Induced Increase in Sweet Solution Intake: Bars represent the mean (±S.E.M.) consumption in grams (g) of a sweet solution prepared with evaporated milk blended with 10% sucrose (S) in groups injected in the intra-NAc shell with (**a**) vehicle (0.9% saline), PD-168077 (0.1 µg), AMPA (0.1 µg), and AMPA + PD-168077 in sated rats; and (**b**) vehicle (0.9% saline), PD-168077, CNQX (0.375 µg), and CNQX + PD-168077. Following a significant one-way ANOVA, Dunnett’s post hoc test was conducted, with * *p* < 0.05, *** *p* < 0.001 indicating significance compared to the vehicle-treated group.

We next examined the role of AMPARs in D4R regulation of palatable solution consumption. One-way ANOVA indicated a significant effect among treatment groups (F(3, 19) = 19.18, *p* < 0.0001). Dunnett’s post hoc test confirmed that intra-NAc shell administration of PD-168077 significantly increased sweet solution intake (*p* = 0.0124), and this effect was prevented by pretreatment with AMPA (an AMPAR agonist). Furthermore, AMPA alone administered to the intra-NAc shell led to a marked decrease in solution consumption (*p* = 0.0008) (Figure 5a).

In contrast, pretreatment with CNQX (an AMPAR antagonist) did not alter the PD-168077-induced increase in solution intake. Interestingly, intra-NAc shell administration of CNQX alone increased solution consumption (*p* = 0.0182) (Figure 5b).

## 4. Discussion

The current findings indicate that intra-NAc shell administration of the D4R agonist PD-168077 significantly enhanced the consumption of a palatable solution, with this effect being effectively reversed by the D4R antagonist L-745870. Furthermore, selective activation of NMDAR also reversed the PD-168077-induced increase in solution intake. AMPAR involvement was similarly confirmed, as pretreatment with AMPA counteracted the effect of PD-168077, and AMPA administration alone led to a notable reduction in solution intake. Interestingly, the combined administration of CNQX with PD-168077 did not reduce intake, and CNQX alone notably increased sweet solution consumption (Table 2).

These findings underscore the complex interplay between dopaminergic and glutamatergic signaling in the NAc shell, where D4R activation appears to facilitate palatable food intake, which is mediated in part by AMPAR and NMDAR activity.

### 4.1. The Activation of D4R in the NAc Shell Increases the Consumption of the Sweet Solution

Overeating often occurs without physiological hunger, driven by the sensory properties of palatable foods, which can activate the brain’s reward system and lead to compulsive eating [33,34]. Eating increases dopamine in the NAc. In the NAc, the medial shell represents food palatability (liking), while both the medial shell and core represent the motivation to eat (wanting) [35,36]. This study focuses on the shell region. Dopamine is vital in the motivation associated with natural stimuli such as food, particularly those rich in sugars and fat, which are potent rewards [2,5].

Numerous studies have elucidated the impact of dopamine in the NAc shell on food consumption. The infusion of the D1R antagonist SCH-23390 facilitates the intake of dietary fat in male Sprague Dawley rats following a high-fat, high-sugar diet, particularly within a 2 h window post-infusion in the rostral region of the NAc shell [37]. The administration of D1 and D2 dopamine antagonists directly into the NAc shell partially attenuated the weight loss and suppression of food intake induced by the appetite suppressant D-norpseudoephedrine [38]. Methylphenidate decreased sucrose consumption in restricted-feeding adult rats. Additionally, histological analyses demonstrated that methylphenidate led to selectively increased binding of dopamine transporter and D2-like receptors in the NAc shell [39]. These findings imply that dopamine receptors play a significant role in regulating food intake.

Since its discovery, D4Rs have been identified as a therapeutically relevant target for treating neuropsychiatric disorders [40]. Recently, their potential has gained attention as a target for various conditions, including cancer, Parkinson’s disease, addiction, eating disorders, erectile dysfunction, and cognitive deficits [5,41]. Despite extensive research on its role in these areas, the relationship between D4Rs and overeating or obesity has received comparatively little focus [42,43].

The results of our study confirm that activation of the D4 receptor (D4R) in the NAc shell significantly influences the consumption of appetitive foods in rats. The administration of PD-168077, a selective D4R agonist, led to a notable increase in the intake of appetitive foods, suggesting that these receptors play a pivotal role in regulating the consumption of palatable solutions.

It is proposed that the consumption of palatable foods promotes dopamine release from the ventral tegmental area (VTA) to the GABAergic neurons of the NAc [44]. This activation of D4Rs, which are presynaptically located in the glutamatergic terminals of NAc neurons [29,45], decreases glutamate release [20] through the inhibition of adenylyl cyclase and the activation of G protein-coupled inwardly rectifying potassium (GIRK) channels [7,10,46,47]. Consequently, this inhibition would prevent the activation of AMPA receptors (AMPAR) and N-methyl-D-aspartate receptors (NMDAR) in the GABAergic neurons of the NAc, leading to their inhibition and an increase in food intake.

Previous studies have shown that inhibiting NAc shell neurons with GABAA agonists and glutamatergic antagonists also enhances food intake [26,48]. Additionally, optogenetic stimulation of glutamatergic fibers within the NAc has been demonstrated to inhibit food consumption [49]. This hyperphagic effect has been similarly observed in the paraventricular nucleus (PVN); our group previously showed that activating D4R in the PVN resulted in increased consumption of standard food in rats subjected to a comparable food restriction regimen [43].

Moreover, a study found that the R7 allele of D4Rs in girls is associated with increased fat and protein intake compared to non-carriers. Those with this allele also tended to decrease their intake of vegetables, eggs, nuts, and bread while increasing their consumption of ice cream (a palatable food) [42]. The presence of the R7 allele of D4Rs has been linked to the overconsumption of both drugs and palatable food, with individuals carrying this allele being more susceptible to overweight and obesity [14,50].

### 4.2. NMDA Reverses the Effect of D4R Activation but Not by the Antagonist AP-5

While NMDA reversed the effect of D4R activation, this treatment did not impact palatable food intake when administered alone. Conversely, the NMDAR antagonist AP-5 also did not influence feeding behavior. Research examining the role of NMDARs in the NAc shell concerning food intake is limited. One study found that the uncompetitive NMDAR antagonist memantine decreases the consumption of palatable food pellets when administered in the NAc shell [51]. However, another study reported that the administration of the NMDAR antagonists MK-801 and AP-5 had no significant effect on food intake in either the NAc shell or core [26].

Research has demonstrated that the effects of pharmacological treatments on food intake are influenced by both the type of food (palatable vs. non-palatable) and the feeding protocol (restricted vs. non-restricted). For instance, treatments involving bilateral microinjection of AMPA or NMDA into the NAc shell significantly and dose-dependently increased standard chow intake over a 4 h period in non-deprived rats [52]. Notably, our research group observed similar findings in the PVN, where NMDA administration mitigated the stimulatory effect of D4Rs on standard food intake. It is proposed that D4R activation presynaptically reduces glutamate release, subsequently diminishing the activation of glutamatergic receptors and inhibiting neuronal activity [43]. If these neurons are inhibitory or anorexigenic, their inhibition would lessen the suppression of food intake, potentially resulting in increased feeding behavior. This interpretation is bolstered by the observation that pretreatment with NMDA in the presence of PD-168077 reverses the effects of PD-168077, indicating a shared underlying pathway.

Electrophysiological studies on pyramidal neurons in the prefrontal cortex have shown that D4R activation reduces evoked currents, excitatory postsynaptic currents (EPSCs), and surface expression of NMDAR through the inhibition of PKA and CaMKII [53]. Additionally, another study indicated that the activation of hD4.7 receptors leads to a significant decrease in the NMDAR/PSD-95 complex, which considerably reduces the expression of NMDAR on the cell membrane [54]. This effect may occur at the presynaptic level, as evidence suggests that NMDAR are present presynaptically in the glutamatergic terminal axons of the NAc shell in adult rats [55]. Furthermore, these NMDAR are found in corticostriatal projections in association with D4R. This arrangement could represent excitatory presynaptic NMDA autoreceptors and inhibitory D4 heteroreceptors that regulate the release of glutamate from corticostriatal axons in the NAc [56]. The colocalization of the D4R and NMDAR in the terminal axons of the NAc suggests a dual role in presynaptic neurotransmitter release in this region.

**Figure 6:**
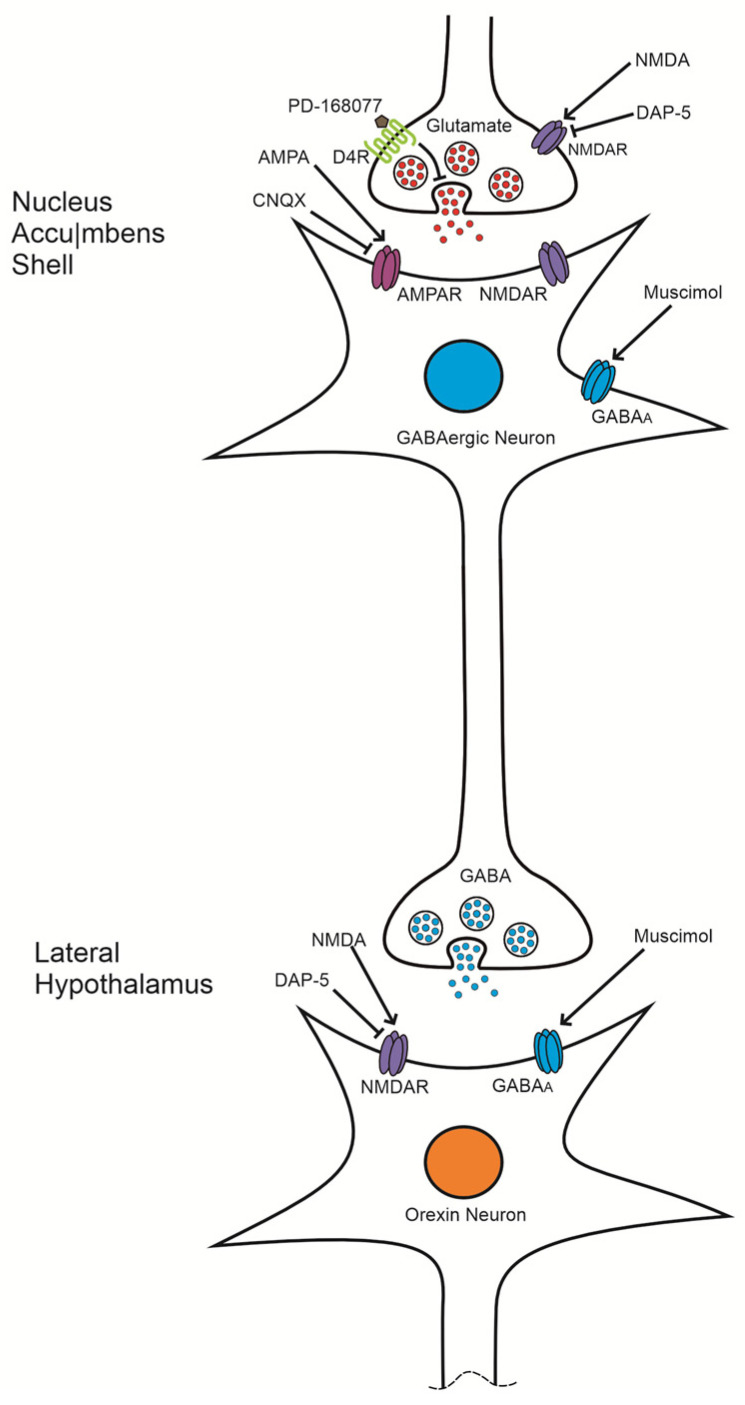
Proposed mechanism of D4 receptor (D4R) activation in regulating palatable food intake: This schematic illustrates the hypothesized pathway through which D4R activation modulates palatable food consumption. Activation of D4Rs is suggested to inhibit glutamate release, which reduces the activity of GABAergic neurons projecting to the lateral hypothalamus (LH). This reduction in GABAergic inhibition allows for the activation of orexinergic neurons, thereby enhancing food intake. A comparable increase in palatable food consumption is observed when AMPA receptors (AMPARs) are blocked by CNQX. In contrast, AMPAR activation is thought to produce the opposite effect, lowering palatable food intake by increasing GABAergic transmission.

The administration of NMDA may increase the synaptic current mediated by NMDAR, counteracting the hypofunction of these receptors induced by D4R activation with PD-168077. By enhancing presynaptic glutamate release, NMDA could counteract the inhibitory effect of D4R, thereby restoring food intake to its basal level. This mechanism may also explain why blocking NMDAR with the antagonist AP-5, in the presence of D4R activation, does not reduce the increase in high-calorie food intake; it would effectively mimic the D4R-induced hypofunction of NMDAR. Collectively, these findings support the role of D4Rs in regulating glutamate release and their relationship with palatable food consumption (Figure 6).

### 4.3. The Effect of D4R Activation Is Reversed by AMPA but Not by the Antagonist CNQX

The results of this study indicate that the effects of D4R activation can be reversed by pretreatment with AMPA; notably, this treatment alone reduces the intake of palatable food. There are very few studies that have administered AMPA centrally. However, injections of AMPA in the NAc shell have been shown to decrease the intake of standard food pellets in deprived rats and palatable solutions (5% sucrose) in non-deprived rats [28]. Our study corroborates these findings, as we observed a similar effect in rats subjected to a food restriction protocol with access to palatable food. Interestingly, like the effect of D4R activation, blockade of AMPAR with CNQX resulted in increased palatable food intake.

Evidence indicates that both AMPAR antagonists, DNQX and CNQX, increase the intake of standard and palatable food when administered locally in the NAc shell [57,58,59,60]. This observation is further supported by the finding that the increase in palatable food intake induced by CNQX, an AMPA receptor antagonist, mirrors the effects seen with D4R activation, likely due to a reduction in neuronal activity within the NAc shell. Additionally, the administration of the GABAA agonist muscimol in the NAc shell has been shown to enhance food intake and elevate c-Fos immunoreactivity—a marker of neuronal activation—in brain regions involved in energy homeostasis, including the PVN, lateral hypothalamus (LH), lateral septum, ventral tegmental area (VTA), and nucleus tractus solitarius (NTS) [26,61]. Notably, the muscimol-induced increase in food intake in the NAc shell is reversed by pretreatment with the NMDA receptor antagonist AP-5 in the LH, underscoring the pivotal role of LH neuron activation in this response [61].

However, pretreatment with CNQX in the presence of PD-168077 did not elevate palatable food intake beyond the levels observed with the individual treatments, suggesting a common pathway in their mechanism of action. Evidence indicates that D4R are predominantly located in the prefrontal cortex (PFC) on pyramidal glutamatergic neurons, including their projections to the striatum [56,62].

Additionally, D4R have been identified presynaptically in the NAc shell, where they modulate excitatory neurotransmission [45]. Based on this, it can be hypothesized that D4R are present presynaptically on glutamatergic terminals projecting from the PFC to the NAc shell. The activation of these receptors could reduce glutamate release and inhibit glutamatergic transmission, a mechanism that has been reported in other regions of the central nervous system, such as the PVN, NAc, and caudoputamen [20,43].

Furthermore, the NAc shell sends inhibitory (GABAergic) projections to orexin-positive neurons in the lateral hypothalamus [63]. Administration of muscimol in the NAc shell has been shown to increase c-Fos expression in these orexin-positive neurons [64,65], indicating a strong association between increased food intake following muscimol administration and heightened activity of orexin-positive neurons in the LH. This suggests that inactivation of NAc shell neurons may disinhibit orexigenic neurons in the LH. Supporting this, several studies have demonstrated that orexin administration in the LH significantly enhances feeding behavior [66,67,68]. Additionally, optogenetic stimulation of the tuberal LH subregion (tLH) produces a marked increase in food intake compared to the anterior LH subregion [69]. Moreover, NMDA administration in the tLH has been found to increase food intake, emphasizing the role of LH activation and glutamatergic transmission in regulating feeding behavior (Figure 6) [70].

Finally, food restriction (FR) has been linked to an increased synaptic incorporation of calcium-permeable AMPARs, potentially enhancing the reward value of highly palatable foods and dopaminergic transmission [22]. Furthermore, FR promotes the insertion of AMPARs into the postsynaptic membranes in regions such as the NAc, hippocampus, and dorsal striatum, thereby modulating glutamatergic synaptic activity [71,72]. This suggests that the food restriction protocol used in this study may have upregulated AMPARs in the NAc shell. Alongside the previously mentioned studies, these findings may provide insights into the mechanisms underlying the observed effects on food intake following the administration of AMPAR drugs.

Thus, the presynaptic regulation of glutamate release reveals a differential role of AMPA and NMDA in the regulation of palatable food intake. The precise mechanisms of these differences warrant further investigation.

## 5. Conclusions

This study provides evidence that the D4R in the NAc shell promotes the consumption of a sweet solution and that this effect depends on glutamatergic transmission. To our knowledge, this is the first study demonstrating that the NAc shell D4R modulates the intake of a palatable sweet solution, suggesting its involvement in hedonic eating. These findings not only advance our understanding of how D4Rs regulate reward-driven food consumption but also highlight D4Rs as a promising pharmacological target for developing new treatments for obesity and binge eating disorders. Specifically, targeting D4Rs could offer an innovative approach to modulate excessive food intake, especially in individuals with impaired satiety responses. Further research is needed to understand the cellular and molecular mechanisms underlying the D4R-mediated intake of sugar-sweetened solutions.

### Future Directions

We propose to introduce a microdialysis probe into the NAc shell, coupled with behavioral measurements of food intake, to activate the D4R while the rat consumes condensed milk. This approach will allow us to quantify dopamine and glutamate levels using high-pressure liquid chromatography (HPLC), helping to corroborate the hypothesis that condensed milk consumption increases dopamine release and that activating D4Rs reduces glutamate release. To determine the presynaptic localization of the D4R in the glutamatergic terminals of the NAc shell, we will conduct an electrophysiological study by measuring miniature excitatory postsynaptic currents (mEPSCs). Additionally, co-immunoprecipitation will be utilized on NAc shell synaptosomes to identify the presynaptic co-localization of NMDAR and D4R. Finally, we will evaluate the effect of the antagonist L-745870 on the D4R in the NAc shell and its influence on condensed milk intake at different doses. This will help determine whether L-745870 blocks the hyperphagic effect induced by D4R activation, suggesting its potential as a therapeutic target in obesity treatment.

## Figures and Tables

**Table 1:** Experimental conditions for evaluating D4R activation, blockade, and its pharmacological modulation by NMDARs and AMPARs in palatable food intake.

1. DR4 Treatments	2. NMDAR Agonist + D4R Agonist	3. NMDAR Antagonist + D4R Agonist	4. AMPAR Agonist + DR4 Agonist	5. AMPAR Antagonist + DR4 Agonist
Saline + Saline	Saline + Saline	Saline + Saline	Saline + Saline	Saline + Saline
Saline + PD-168077	Saline + PD-168077	Saline + PD-168077	Saline + PD-168077	Saline + PD-168077
Saline + L-745870	NMDA + PD-168077	AP5 + PD-168077	AMPA + PD-168077	CNQX + PD-168077
L-745870+ PD-168077	Saline + NMDA	Saline + AP5	Saline + AMPA	Saline + CNQX

**Table 2:** Summary of the main highlights of the current study.

Intra NAc shell administration of the D4R agonist, PD-168077, stimulates the intake of a sweet solution. Intra NAc shell co-administration of NMDA, but not of AP5, reverts the PD-16877-induced sweet solution intake. Intra NAc shell co-administration of AMPA, but not CNQX, reverts the PD-16977-induced sweet solution intake. Intra NAc shell administration of AMPA alone inhibits the intake of a sweet solution.

## Data Availability

Data are contained within the article.

## References

[1] Baik J.H. (2021). Dopaminergic Control of the Feeding Circuit. Endocrinol. Metab..

[2] Volkow N.D., Wang G.J., Baler R.D. (2011). Reward, Dopamine and the Control of Food Intake: Implications for Obesity. Trends Cogn. Sci..

[3] Krause M., German P.W., Taha S.A., Fields H.L. (2010). A Pause in Nucleus Accumbens Neuron Firing Is Required to Initiate and Maintain Feeding. J. Neurosci..

[4] Fetterly T.L., Catalfio A.M., Ferrario C.R. (2024). Effects of Junk-Food on Food-Motivated Behavior and Nucleus Accumbens Glutamate Plasticity; Insights into the Mechanism of Calcium-Permeable AMPA Receptor Recruitment. Neuropharmacology.

[5] Di Ciano P., Grandy D.K., Le Foll B. (2014). Dopamine D4 Receptors in Psychostimulant Addiction. Advances in Pharmacology.

[6] Mietlicki-Baase E.G., Ortinski P.I., Reiner D.J., Sinon C.G., McCutcheon J.E., Pierce R.C., Roitman M.F., Hayes M.R. (2014). Glucagon-like Peptide-1 Receptor Activation in the Nucleus Accumbens Core Suppresses Feeding by Increasing Glutamatergic AMPA/Kainate Signaling. J. Neurosci..

[7] Rondou P., Haegeman G., Van Craenenbroeck K. (2010). The Dopamine D4 Receptor: Biochemical and Signalling Properties. Cell. Mol. Life Sci. CMLS.

[8] Rada P., Avena N.M., Hoebel B.G. (2005). Daily Bingeing on Sugar Repeatedly Releases Dopamine in the Accumbens Shell. Neuroscience.

[9] Volkow N.D., Michaelides M., Baler R. (2019). The Neuroscience of Drug Reward and Addiction. Physiol. Rev..

[10] Oak J.N., Oldenhof J., Van Tol H.H.M. (2000). The Dopamine D4 Receptor: One Decade of Research. Eur. J. Pharmacol..

[11] Kalyanasundar B., Perez C.I., Luna A., Solorio J., Moreno M.G., Elias D., Simon S.A., Gutierrez R. (2015). D1 and D2 Antagonists Reverse the Effects of Appetite Suppressants on Weight Loss, Food Intake, Locomotion, and Rebalance Spiking Inhibition in the Rat NAc Shell. J. Neurophysiol..

[12] Kim K.S., Yoon Y.R., Lee H.J., Yoon S., Kim S.Y., Shin S.W., An J.J., Kim M.S., Choi S.Y., Sun W. (2010). Enhanced Hypothalamic Leptin Signaling in Mice Lacking Dopamine D2 Receptors. J. Biol. Chem..

[13] Johnson P.M., Kenny P.J. (2010). Dopamine D2 Receptors in Addiction-like Reward Dysfunction and Compulsive Eating in Obese Rats. Nat. Neurosci..

[14] Botticelli L., Micioni Di Bonaventura E., Del Bello F., Giorgioni G., Piergentili A., Romano A., Quaglia W., Cifani C., Micioni Di Bonaventura M.V. (2020). Underlying Susceptibility to Eating Disorders and Drug Abuse: Genetic and Pharmacological Aspects of Dopamine D4 Receptors. Nutrients.

[15] Kaur G., Kulkarni S.K. (2002). Studies on Modulation of Feeding Behavior by Atypical Antipsychotics in Female Mice. Prog. Neuropsychopharmacol. Biol. Psychiatry.

[16] Lee M.D., Clifton P.G. (2002). Meal Patterns of Free Feeding Rats Treated with Clozapine, Olanzapine, or Haloperidol. Pharmacol. Biochem. Behav..

[17] Cruz-Trujillo R., Tejas-Juárez J.G., Mancilla-Díaz J.M., Escartín Pérez R.E. (2020). Evaluación Del Bloqueo de Los Receptores Dopaminérgicos D4 En El Núcleo Accumbens Sobre La Motivación Por El Alimento Palatable. Espacio I+ D Innovación más desarrollo.

[18] López-Alonso V.E., Hernández-Correa S., Escobar C., Escartín-Pérez R.E., Mancilla-Díaz J.M., Díaz-Urbina D. (2023). The Central Blockade of the Dopamine DR4 Receptor Decreases Sucrose Consumption by Modifying the Microstructure of Drinking Behavior in Male Rats. IBRO Neurosci. Rep..

[19] Cruz-Trujillo R., Avalos-Fuentes A., Rangel-Barajas C., Paz-Bermúdez F., Sierra A., Escartín-Perez E., Aceves J., Erlij D., Florán B. (2013). D3 Dopamine Receptors Interact with Dopamine D1 but Not D4 Receptors in the GABAergic Terminals of the SNr of the Rat. Neuropharmacology.

[20] González S., Rangel-Barajas C., Peper M., Lorenzo R., Moreno E., Ciruela F., Borycz J., Ortiz J., Lluís C., Franco R. (2012). Dopamine D 4 Receptor, but Not the ADHD-Associated D 4.7 Variant, Forms Functional Heteromers with the Dopamine D 2S Receptor in the Brain. Mol. Psychiatry.

[21] Khan Z.U., Gutiérrez A., Martín R., Peñafiel A., Rivera A., De La Calle A. (1998). Differential Regional and Cellular Distribution of Dopamine D2-like Receptors: An Immunocytochemical Study of Subtype-Specific Antibodies in Rat and Human Brain. J. Comp. Neurol..

[22] Carr K.D. (2020). Homeostatic Regulation of Reward via Synaptic Insertion of Calcium-Permeable AMPA Receptors in Nucleus Accumbens. Physiol. Behav..

[23] Dong X., Li S., Kirouac G.J. (2017). Collateralization of Projections from the Paraventricular Nucleus of the Thalamus to the Nucleus Accumbens, Bed Nucleus of the Stria Terminalis, and Central Nucleus of the Amygdala. Brain Struct. Funct..

[24] Stuber G.D., Sparta D.R., Stamatakis A.M., Van Leeuwen W.A., Hardjoprajitno J.E., Cho S., Tye K.M., Kempadoo K.A., Zhang F., Deisseroth K. (2011). Excitatory Transmission from the Amygdala to Nucleus Accumbens Facilitates Reward Seeking. Nature.

[25] Kelley A.E., Baldo B.A., Pratt W.E., Will M.J. (2005). Corticostriatal-Hypothalamic Circuitry and Food Motivation: Integration of Energy, Action and Reward. Physiol. Behav..

[26] Maldonado-lrizarry C.S., Swanson C.J., Kelley A.E. (1995). Glutamate Receptors in the Nucleus Accumbens Shell Control Feeding Behavior via the Lateral Hypothalamus. J. Neurosci..

[27] Mena J.D., Selleck R.A., Baldo B.A. (2013). Mu-Opioid Stimulation in Rat Prefrontal Cortex Engages Hypothalamic Orexin/Hypocretin-Containing Neurons, and Reveals Dissociable Roles of Nucleus Accumbens and Hypothalamus in Cortically Driven Feeding. J. Neurosci..

[28] Stratford T.R., Swanson C.J., Kelley A. (1998). Specific Changes in Food Intake Elicited by Blockade or Activation of Glutamate Receptors in the Nucleus Accumbens Shell. Behav. Brain Res..

[29] Bonaventura J., Quiroz C., Cai N.S., Rubinstein M., Tanda G., Ferré S. (2017). Key Role of the Dopamine D4 Receptor in the Modulation of Corticostriatal Glutamatergic Neurotransmission. Sci. Adv..

[30] Ferré S., Belcher A.M., Bonaventura J., Quiroz C., Sánchez-Soto M., Casadó-Anguera V., Cai N.S., Moreno E., Boateng C.A., Keck T.M. (2022). Functional and Pharmacological Role of the Dopamine D4 Receptor and Its Polymorphic Variants. Front. Endocrinol..

[31] Cortés-Salazar F., Suárez Ortíz J.O., Cendejas Trejo N.M., Mancilla-Díaz J.M., López-Alonso V.E., Escartín-Pérez R.E. (2014). Effects of CB1 Cannabinoid Receptor Activation in the Nucleus Accumbens Shell on Feeding Behavior. Acta Colomb. Psicol..

[32] Cortés-Salazar F., Mancilla-Díaz J.M., López-Alonso V.E., González-Hernández B., Escartín-Pérez R.E. (2014). Relationship between CB1 Receptors in the Nucleus Accumbens Shell and the Hedonic Value of Food. Recent Hispanic Psychological Research on Feeding Behavior and HIV Patients.

[33] Fletcher P.C., Kenny P.J. (2018). Food Addiction: A Valid Concept?. Neuropsychopharmacology.

[34] Fontana C., Vitolo M.R., Campagnolo P.D.B., Mattevi V.S., Genro J.P., Almeida S. (2015). DRD4 and SLC6A3 Gene Polymorphisms Are Associated with Food Intake and Nutritional Status in Children in Early Stages of Development. J. Nutr. Biochem..

[35] Castro D.C., Berridge K.C. (2014). Opioid Hedonic Hotspot in Nucleus Accumbens Shell: Mu, Delta, and Kappa Maps for Enhancement of Sweetness “Liking” and “Wanting”. J. Neurosci..

[36] Durst M., Könczöl K., Balázsa T., Eyre M.D., Tóth Z.E. (2019). Reward-Representing D1-Type Neurons in the Medial Shell of the Accumbens Nucleus Regulate Palatable Food Intake. Int. J. Obes..

[37] Joshi A., Kool T., Diepenbroek C., Koekkoek L.L., Eggels L., Kalsbeek A., Mul J.D., Barrot M., la Fleur S.E. (2021). Dopamine D1 Receptor Signalling in the Lateral Shell of the Nucleus Accumbens Controls Dietary Fat Intake in Male Rats. Appetite.

[38] Kalyanasundar B., Perez C.I., Arroyo B., Moreno M.G., Gutierrez R. (2020). The Appetite Suppressant D-Norpseudoephedrine (Cathine) Acts via D1/D2-Like Dopamine Receptors in the Nucleus Accumbens Shell. Front. Neurosci..

[39] Bello N.T., Hajnal A. (2006). Acute Methylphenidate Treatments Reduce Sucrose Intake in Restricted-Fed Bingeing Rats. Brain Res. Bull..

[40] Giorgioni G., Del Bello F., Pavletić P., Quaglia W., Botticelli L., Cifani C., Micioni Di Bonaventura E., Micioni Di Bonaventura M.V., Piergentili A. (2021). Recent Findings Leading to the Discovery of Selective Dopamine D4 Receptor Ligands for the Treatment of Widespread Diseases. Eur. J. Med. Chem..

[41] Lindsley C.W., Hopkins C.R. (2017). Return of D4 Dopamine Receptor Antagonists in Drug Discovery. J. Med. Chem..

[42] Silveira P.P., Portella A.K., Kennedy J.L., Gaudreau H., Davis C., Steiner M., Soares C.N., Matthews S.G., Sokolowski M.B., Dubé L. (2014). Association between the Seven-Repeat Allele of the Dopamine-4 Receptor Gene (DRD4) and Spontaneous Food Intake in Pre-School Children. Appetite.

[43] Tejas-Juárez J.G., Cruz-Martínez A.M., López-Alonso V.E., García-Iglesias B., Mancilla-Díaz J.M., Florán-Garduño B., Escartín-Pérez R.E. (2014). Stimulation of Dopamine D4 Receptors in the Paraventricular Nucleus of the Hypothalamus of Male Rats Induces Hyperphagia: Involvement of Glutamate. Physiol. Behav..

[44] Yager L.M., Garcia A.F., Wunsch A.M., Ferguson S.M. (2015). The Ins and Outs of the Striatum: Role in Drug Addiction. Neuroscience.

[45] Svingos A.L., Periasamy S., Pickel V.M. (2000). Presynaptic Dopamine D4 Receptor Localization in the Rat Nucleus Accumbens Shell. Synapse.

[46] Pillai G., Brown N.A., McAllister G., Milligan G., Seabrook G.R. (1998). Human D2 and D4 Dopamine Receptors Couple through Βγ G-Protein Subunits to Inwardly Rectifying K+ Channels (GIRK1) in a Xenopus Oocyte Expression System: Selective Antagonism by L-741,626 and L-745,870 Respectively. Neuropharmacology.

[47] Wedemeyer C., Goutman J.D., Avale M.E., Franchini L.F., Rubinstein M., Calvo D.J. (2007). Functional Activation by Central Monoamines of Human Dopamine D4 Receptor Polymorphic Variants Coupled to GIRK Channels in Xenopus Oocytes. Eur. J. Pharmacol..

[48] Stratford T.R., Kelley A.E. (1997). GABA in the Nucleus Accumbens Shell Participates in the Central Regulation of Feeding Behavior. J. Neurosci..

[49] Prado L., Luis-Islas J., Sandoval O.I., Puron L., Gil M.M., Luna A., Arias-García M.A., Galarraga E., Simon S.A., Gutierrez R. (2016). Activation of Glutamatergic Fibers in the Anterior NAc Shell Modulates Reward Activity in the aNAcSh, the Lateral Hypothalamus, and Medial Prefrontal Cortex and Transiently Stops Feeding. J. Neurosci..

[50] Levitan R.D., Jansen P., Wendland B., Tiemeier H., Jaddoe V.W., Silveira P.P., Kennedy J.L., Atkinson L., Fleming A., Sokolowski M. (2017). A DRD4 Gene by Maternal Sensitivity Interaction Predicts Risk for Overweight or Obesity in Two Independent Cohorts of Preschool Children. J. Child Psychol. Psychiatry.

[51] Smith K.L., Rao R.R., Velázquez-Sánchez C., Valenza M., Giuliano C., Everitt B.J., Sabino V., Cottone P. (2015). The Uncompetitive N-Methyl-D-Aspartate Antagonist Memantine Reduces Binge-Like Eating, Food-Seeking Behavior, and Compulsive Eating: Role of the Nucleus Accumbens Shell. Neuropsychopharmacology.

[52] Echo J.A., Lamonte N., Christian G., Znamensky V., Ackerman T.F., Bodnar R.J. (2001). Excitatory Amino Acid Receptor Subtype Agonists Induce Feeding in the Nucleus Accumbens Shell in Rats: Opioid Antagonist Actions and Interactions with μ-Opioid Agonists. Brain Res..

[53] Wang X., Zhong P., Gu Z., Yan Z. (2003). Regulation of NMDA Receptors by Dopamine D4 Signaling in Prefrontal Cortex. J. Neurosci..

[54] Qin L., Liu W., Ma K., Wei J., Zhong P., Cho K., Yan Z. (2016). The ADHD-Linked Human Dopamine D4 Receptor Variant D4.7 Induces over-Suppression of NMDA Receptor Function in Prefrontal Cortex. Neurobiol. Dis..

[55] Gracy K.N., Svingos A.L., Pickel V.M. (1997). Dual Ultrastructural Localization of μ-Opioid Receptors and NMDA-Type Glutamate Receptors in the Shell of the Rat Nucleus Accumbens. J. Neurosci..

[56] Tarazi F.I., Campbell A., Yeghiayan S.K., Baldessarini R.J. (1998). Localization of Dopamine Receptor Subtypes in Corpus Striatum and Nucleus Accumbens Septi of Rat Brain: Comparison of D1-, D2- and D4-like Receptors. Neuroscience.

[57] Faure A., Reynolds S.M., Richard J.M., Berridge K.C. (2008). Mesolimbic Dopamine in Desire and Dread: Enabling Motivation to Be Generated by Localized Glutamate Disruptions in Nucleus Accumbens. J. Neurosci..

[58] Reynolds S.M., Berridge K.C. (2008). Emotional Environments Retune the Valence of Appetitive versus Fearful Functions in Nucleus Accumbens. Nat. Neurosci..

[59] Reynolds S.M., Berridge K.C. (2003). Glutamate Motivational Ensembles in Nucleus Accumbens: Rostrocaudal Shell Gradients of Fear and Feeding. Eur. J. Neurosci..

[60] Urstadt K.R., Coop S.H., Banuelos B.D., Stanley B.G. (2013). Behaviorally Specific versus Non-Specific Suppression of Accumbens Shell-Mediated Feeding by Ipsilateral versus Bilateral Inhibition of the Lateral Hypothalamus. Behav. Brain Res..

[61] Stratford T.R., Kelley A.E. (1999). Evidence of a Functional Relationship between the Nucleus Accumbens Shell and Lateral Hypothalamus Subserving the Control Oil Feeding Behavior. J. Neurosci..

[62] Lauzon N.M., Laviolette S.R. (2010). Dopamine D 4-Receptor Modulation of Cortical Neuronal Network Activity and Emotional Processing: Implications for Neuropsychiatric Disorders. Behav. Brain Res..

[63] Perez-Leighton C., Little M.R., Grace M., Billington C., Kotz C.M. (2017). Orexin Signaling in Rostral Lateral Hypothalamus and Nucleus Accumbens Shell in the Control of Spontaneous Physical Activity in High- and Low-Activity Rats. Am. J. Physiol. Regul. Integr. Comp. Physiol..

[64] Baldo B.A., Gual-Bonilla L., Sijapati K., Daniel R.A., Landry C.F., Kelley A.E. (2004). Activation of a Subpopulation of Orexin/Hypocretin-Containing Hypothalamic Neurons by GABAA Receptor-Mediated Inhibition of the Nucleus Accumbens Shell, but Not by Exposure to a Novel Environment. Eur. J. Neurosci..

[65] Zheng H., Corkern M., Stoyanova I., Patterson L.M., Tian R., Berthoud H.R. (2003). Appetite-Inducing Accumbens Manipulation Activates Hypothalamic Orexin Neurons and Inhibits POMC Neurons. Am. J. Physiol.-Regul. Integr. Comp. Physiol..

[66] Barson J.R., Leibowitz S.F. (2017). Orexin/Hypocretin System: Role in Food and Drug Overconsumption. International Review of Neurobiology.

[67] Thorpe A.J., Mullett M.A., Wang C., Kotz C.M. (2003). Regional, Metabolic, and Circadian Specificity of Lateral Hypothalamic Orexin A Feeding Stimulation. Am. J. Physiol.-Regul. Integr. Comp. Physiol..

[68] Imperatore R., Palomba L., Cristino L. (2017). Role of Orexin-A in Hypertension and Obesity. Curr. Hypertens. Rep..

[69] Urstadt K.R., Berridge K.C. (2020). Optogenetic Mapping of Feeding and Self-Stimulation within the Lateral Hypothalamus of the Rat. PLoS ONE.

[70] Duva M.A., Tomkins E.M., Moranda L.M., Kaplan R., Sukhaseum A., Stanley B.G. (2005). Origins of Lateral Hypothalamic Afferents Associated with N-Methyl-d-Aspartic Acid-Elicited Eating Studied Using Reverse Microdialysis of NMDA and Fluorogold. Neurosci. Res..

[71] Ouyang J., Carcea I., Schiavo J.K., Jones K.T., Rabinowitsch A., Kolaric R., Cabeza de Vaca S., Froemke R.C., Carr K.D. (2017). Food Restriction Induces Synaptic Incorporation of Calcium-Permeable AMPA Receptors in Nucleus Accumbens. Eur. J. Neurosci..

[72] Campanelli F., Laricchiuta D., Natale G., Marino G., Calabrese V., Picconi B., Petrosini L., Calabresi P., Ghiglieri V. (2021). Long-Term Shaping of Corticostriatal Synaptic Activity by Acute Fasting. Int. J. Mol. Sci..

